# Functional selective FPR1 signaling in favor of an activation of the neutrophil superoxide generating NOX2 complex

**DOI:** 10.1002/JLB.2HI0520-317R

**Published:** 2020-10-11

**Authors:** Simon Lind, Claes Dahlgren, Rikard Holmdahl, Peter Olofsson, Huamei Forsman

**Affiliations:** ^1^ Department of Rheumatology and Inflammation Research Institute of Medicine University of Gothenburg Gothenburg Sweden; ^2^ Medical Inflammation Research, Department of Medical Biochemistry and Biophysics Karolinska Institute Stockholm Sweden

**Keywords:** Biased signaling, Chemotaxis, Formyl peptide receptors, NADPH‐oxidase, Neutrophils, Small compounds

## Abstract

The formyl peptide receptors FPR1 and FPR2 are abundantly expressed by neutrophils, in which they regulate proinflammatory tissue recruitment of inflammatory cells, the production of reactive oxygen species (ROS), and resolution of inflammatory reactions. The unique dual functionality of the FPRs makes them attractive targets to develop FPR‐based therapeutics as novel anti‐inflammatory treatments. The small compound RE‐04‐001 has earlier been identified as an inducer of ROS in differentiated HL60 cells but the precise target and the mechanism of action of the compound was has until now not been elucidated. In this study, we reveal that RE‐04‐001 specifically targets and activates FPR1, and the concentrations needed to activate the neutrophil NADPH‐oxidase was very low (EC_50_ ∼1 nM). RE‐04‐001 was also found to be a neutrophil chemoattractant, but when compared to the prototype FPR1 agonist N‐formyl‐Met‐Leu‐Phe (fMLF), the concentrations required were comparably high, suggesting that signaling downstream of the RE‐04‐001‐activated‐FPR1 is functionally selective. In addition, the RE‐04‐001‐induced response was strongly biased toward the PLC‐PIP_2_‐Ca^2+^ pathway and ERK1/2 activation but away from β‐arrestin recruitment. Compared to the peptide agonist fMLF, RE‐04‐001 is more resistant to inactivation by the MPO‐H_2_O_2_‐halide system. In summary, this study describes RE‐04‐001 as a novel small molecule agonist specific for FPR1, which displays a biased signaling profile that leads to a functional selective activating of human neutrophils. RE‐04‐001 is, therefore, a useful tool, not only for further mechanistic studies of the regulatory role of FPR1 in inflammation in vitro and in vivo, but also for developing FPR1‐specific drug therapeutics.

## Graphical Abstract

RE‐04‐001 is a novel small molecule agonist specific for FPR1, which displays a biased signaling profile that leads to a functional selective activating of human neutrophils

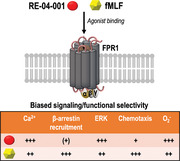

**Abbreviations**
[Ca^2+^]_i_: intracellular calcium
CL: chemiluminescence
CPM: counts per minute
FFAR2: free fatty acid receptor 2
fMLF: N‐formyl‐Met‐Leu‐Phe
FPR: formyl peptide receptor
GPCR: G protein‐coupled receptor
MPO: myeloperoxidase
NOX2 complex: NADPH‐oxidase complex
PAF: platelet‐activating factor
PAFR: platelet‐activating factor receptor
ROS: reactive oxygen species

## 1 INTRODUCTION

Neutrophils express several G protein‐coupled receptors (GPCRs) that regulate cell functions and fine‐tune inflammatory reactions.^1^, ^2^ Among these receptors, the chemoattractant formyl peptide receptors (FPR1 and FPR2) have gained much interest over the years and they have been extensively studied by researchers both in academia and in the pharmaceutical industry.^3^, ^4^, ^5^ FPR1 and FPR2 are strongly associated with the progression, as well as the resolution of inflammatory reactions, initiated by microbial infections and/or aseptic tissue injuries.^6^, ^7^ The FPRs recognize not only microbial pathogen associated molecular patterns and host‐derived danger signals in the form of formylated peptides, but also numerous nonformylated peptides/proteins/lipopeptides and other molecules such as small compounds and peptidomimetics.^3^, ^8^, ^9^ FPR1 and FPR2 exhibit a large overall amino acid sequence similarity with a high degree of identity in the cytosolic parts and a lower degree in the extracellular domains.^4^ This suggests that the two receptors differ more when it comes to ligand binding than in the intracellular signals transmitted. Nevertheless, many agonists cross activate the two receptors although there are a few reported that are highly specific for one or the other of the two receptors.^9^ The downstream signals generated by agonists of FPRs regulate neutrophil directional migration (chemotaxis), mobilization of adhesion molecules to the cell surface, and secretion of inflammatory mediators, including proteolytically active proteases. Another feature of FPR agonists is also the activation of the electron transporting NADPH‐oxidase complex type 2 (the NOX2 complex) with the capacity to produce superoxide anions (O_2_^−^) that secondarily generates other reactive oxygen species (ROS).^10^

Activation of neutrophils is essential for defense against microbes and for clearance of harmful tissue debris, but also to limit further neutrophil recruitment and facilitate tissue repair. Thus, these dual functions need to be tightly controlled through the different phases of inflammation. The effect of ROS shows a similar type of complex role when affecting different types of inflammation. ROS released in high quantities from neutrophils is generally regarded as driving acute inflammation. However, it is also clear that ROS could dampen inflammation, an effect more likely to operate in the resolution phases.^11^, ^12^, ^13^ Hence, in light of this complex and so far not completely understood role of FPR and ROS regulation, our accumulated research proposes a regulatory role of ROS produced by the NADPH‐oxidase in many cellular processes.^14^, ^15^ Patients, as well as experimental animals, with chronic granulomatous disease, lacking the ability to generate ROS, suffer not only from severe microbial infections, but also from a variety of inflammatory complications indicative of important functions of ROS in the mechanisms that control inflammation.^16^, ^17^, ^18^ The importance of ROS in the regulation of inflammation also gains support from earlier studies in which we through positional cloning of a disease‐linked genetic polymorphism, have identified *Ncf1* (encoding for the p47^phox^ subunit of the NADPH‐oxidase complex) as a disease‐associated gene^19^ and the molecular basis being linked to a compromised ROS production.^20^ Similarly, polymorphism of *Ncf1* plays a role in human autoimmune diseases,^21^, ^22^ and it has been shown to be of importance for disease severity of arthritis, psoriasis, colitis, and lupus in animal models (reviewed in Holmdahl et al. and Urbonaviciute et al.^14^, ^23^). Hence, it is apparent from both pharmacologic and genetic deletion studies, that FPRs have multiple roles in diseases conditions associated with a dysregulated inflammation. Mice deficient in individual FPRs show not only an increased susceptibility to microbial infections but also a delayed tissue repair.^7^, ^24^, ^25^ In addition, a recent study has elegantly demonstrated that activation of FPRs improves cardiac function in a post myocardial infarction model,^26^ suggesting an anti‐inflammatory/pro‐resolving role of FPR agonists.

The introduction of the biased GPCR signaling concept rapidly became the starting point not only for more detailed characterization of known GPCR agonists but also for the search for new biased GPCR agonists that could be used to develop drug candidates.^27^, ^28^ The concept of biased signaling or functional selectivity describes that different ligands for a given receptor can stabilize receptor in different conformations allowing distinct signaling pathways with different functional activities. The concept has been shown to be valid also for FPR2 as illustrated by the downstream signaling by FPR2‐specific agonistic lipopeptides/pepducins, peptidomimetics, as well as by formylated peptides generated by virulent Staphylococcus *aureus* bacteria.^29^, ^30^, ^31^ These biased FPR2 agonists are potent triggers of a rise in intracellular calcium ([Ca^2+^]_i_) and a release of superoxide through the NADPH‐oxidase but in contrast to earlier described FPR2 agonists, they lack the ability to recruit β‐arrestin and induce chemotaxis.^29^, ^30^, ^31^ Due to the similarities between FPR1 and FPR2 it is reasonable to assume that FPR1 also can be stabilized in a conformation that opens for one signaling pathway downstream of the receptor but not for another. This assumption gains support from a study showing that selective formylpeptide analogues can discriminate between different biologic responses, being able to trigger chemotaxis but not activate the superoxide generating neutrophil NADPH‐oxidase.^32^

In attempt to identify novel ROS activators, we have earlier screened libraries of drug‐compatible small compounds and identified a number of hits belonging to different structural classes.^33^ Among these hits, one lead compound has been further developed as a novel compound class of structures (patent WO2012127214; further developed into a new subclass of compounds represented by the FPR agonist RE‐04‐001). In the hit validation program and lead development, RE‐04‐001 was initially shown to trigger ROS production in differentiated neutrophil‐like HL60 cells with an activation profile that is very similar to well‐characterized FPR agonists. Based on this observation we hypothesized that RE‐04‐001 could be an FPR agonist. Accordingly, a detailed characterization of the compound was subsequently performed, and we now show that RE‐04‐001 is a specific FPR1 agonist inducing a functional selective neutrophil response. This functional selectivity was closely linked to a biased signaling feature in favor of ERK1/2 phosphorylation and rise of [Ca^2+^]_i_ together with an inability to recruit β‐arrestin. The biased agonistic profile of RE‐04‐001, being a potent activator of ROS production, suggests that RE‐04‐001 could serve as a valuable representative novel compound for further mechanistic studies designed to dissect the contribution of different FPR1‐mediated functions in inflammation associated diseases as well as potential therapeutic agent.

## 2 METHODS

### 2.1 Ethics statement

This study, conducted at the Sahlgrenska Academy in Sweden, includes peripheral blood and from buffy coats obtained from the blood bank at Sahlgrenska University Hospital, Gothenburg, Sweden. According to the Swedish legislation section code 4§ 3p SFS 2003:460 (*Lag om etikprövning av forskning som avser människor*), no ethical approval was needed because the blood samples were provided anonymously and cannot be traced back to a specific donor.

### 2.2 Chemicals and reagents

The compound RE‐04‐001, with structure related to the class of compounds known as quinolones that were described in the patent application WO 2012/127214 and reported earlier in screening studies.^33^ For intellectual property reasons, the chemical structure of RE‐04‐001 is not disclosed. For more information about RE‐04‐001 and to make it possible to reproduce the data presented herein, the compound will be provided to other researchers under a material transfer agreement (contact person: Peter Olofsson).

Dextran T500 was obtained from Pharmacocosmos (Holbaek, Denmark), Ficoll‐Paque was from GE Healthcare Bio‐Science AB (Uppsala, Sweden), and Fura‐2‐AM was from Life Technologies Europe (Stockholm, Sweden). RPMI 1640 culture medium without phenol red was purchased from PAA Laboratories GmbH (Pasching, Austria). Isoluminol, N‐formyl‐Met‐Leu‐Phe (fMLF), cetyltrimethylammonium bromide (CTAB), o‐Phenylenediamine (OPD), EGTA, DMSO, platelet‐activating factor (PAF), BSA, and Latrunculin A were obtained from Sigma‐Aldrich (St. Louis, MO, USA). HRP was purchased from Boehringer‐Mannheim (Mannheim, Germany). TNFα and IL8 were from R&D Systems (Minneapolis, MN, USA). PAF was from Calbiochem (San Diego, CA, USA). The FPR2 agonist WKYMVM was synthesized and purified by HPLC by Alta Bioscience (University of Birmingham, Birmingham, United Kingdom). The FPR2‐specific antagonist PBP_10_ was synthesized by CASLO Laboratory (Lyngby, Denmark) and the FPR1‐specific inhibitor (an inverse agonist) cyclosporin H was kindly provided by Novartis Pharma (Basel, Switzerland). The Gαq inhibitor YM‐254890 was purchased from Wako Chemicals (Neuss, Germany). Myeloperoxidase (MPO) and the phenylacetamide compound (S)‐2‐(4‐chlorophenyl)‐3,3‐dimethyl‐N‐(5‐phenylthiazol‐2‐yl)butanamide (Cmp58) was obtained from Calbiochem‐Merck Millipore (Billerica, MA, USA). Compound 43 was from Tocris Bioscience (Bristol, United Kingdom). The Act‐389949 compound,^34^ synthesized by Ramidus AB (Lund, Sweden) is a generous gift from ProNoxis AB (Lund, Sweden). The receptor agonists and antagonists were dissolved in DMSO to a concentration of 10^−2^ M and stored at −80°C until use. Further dilutions were made in Krebs‐Ringer phosphate buffer containing glucose (10 mM), Ca^2+^ (1 mM), and Mg^2+^ (1.5 mM) (KRG; pH 7.3), giving a final concentration of DMSO for all agonists below 0.001%.

### 2.3 Isolation of human neutrophils and culture of neutrophil‐like HL‐60 cells

Neutrophil granulocytes were isolated from peripheral blood or buffy coats obtained from healthy adults.^35^, ^36^ After dextran sedimentation at 1 ×*g*, hypotonic lysis of the remaining erythrocytes, and centrifugation on a Ficoll‐Paque gradient, the neutrophils were washed and resuspended (1 × 10^7^/ml) in KRG. The cells were stored on melting ice until used. The purity of the neutrophil preparations was routinely >90%.

HL60 cells were cultured under sterile conditions at 37°C in 5% CO_2_ in RPMI 1640 medium supplemented with 10% FCS, 2 mM L‐glutamine, 1 mM sodium pyruvate, 100 units/ml penicillin, and 100 µg/ml streptomycin (RPMI 1640 complete medium). Cells were cultured at a density of 2 × 10^5^ cells/ml in tissue culture flasks (75 cm^2^) and differentiated toward a nonadherent neutrophil‐like phenotype by incubation with 1% DMSO for 5 d. Cells were washed and resuspended to 10^6^/ml in KRG, stored on ice until use on day 5 after start of the differentiation.

### 2.4 Calcium mobilization

Neutrophils at a density of 5 × 10^7^ cells/ml in KRG without Ca^2+^ supplemented with 0.1% BSA were loaded with Fura‐2‐AM (5 µM) for 30 min in the dark at room temperature. The cells were then diluted 1:1 in RPMI 1640 culture medium without phenol red and centrifuged at 900 rpm ×*g*. Finally, the cells were washed once with KRG and resuspended in the same buffer to a density of 2 × 10^7^/ml. Calcium measurements were carried out in a PerkinElmer fluorescence spectrophotometer (LC50, Perkin Elmer, Waltham, MA, USA), with excitation wavelengths of 340 nm and 380 nm, an emission wavelength of 509 nm, and slit widths of 5 nm and 10 nm, respectively. The transient rise in [Ca^2+^]_i_ is presented as the ratio of fluorescence intensities (340/380 nm) detected.

### 2.5 Neutrophil NADPH‐oxidase activity

Neutrophil O_2_^−^ production was determined using an isoluminol‐enhanced chemiluminescence (CL) system (details are given in Dahlgren et al.^37^). The CL activity was measured in a six‐channel Biolumat LB 9505 (Berthold Co., Wildbad, Germany) using disposable 4 ml polypropylene tubes with a 1 ml reaction mixture. Tubes containing isoluminol (2 × 10^−5^ M), HRP (2 units/ml), and neutrophils (10^5^/ml) were equilibrated for 5 min at 37°C, after which 0.1 ml of stimuli was added and the superoxide production, measured as light emission (counts per minute, CPM) over time. Representative kinetics were presented in the figures as abscissa, time (min); ordinate, O_2_^−^ production (Mega CPM).

### 2.6 Treatment of FPR agonists with MPO‐H_2_O_2_

Different peptide or small compound FPR agonists were incubated with purified MPO (1 µg/ml) at ambient temperature for 5 min before the addition of H_2_O_2_ (10 µM final concentration), and incubation was continued for another 10 min at ambient temperature to allow peptide oxidation. The remaining activity of the agonists after MPO‐H_2_O_2_‐halide oxidation was determined through the potential of agonist to trigger ROS release from neutrophils. The control agonists were incubated at the same concentration in KRG but with no addition of MPO and H_2_O_2_.

### 2.7 Chemotaxis assay

Neutrophil migration was determined by a Boyden chamber technique using 96‐well microplate chemotaxis chambers containing polycarbonate filters with 3 µm pores (Chemo‐Tx; Neuro Probe, Inc., Gaithersburg, MD, USA) according to manufacturer's instructions. In short, RE‐04‐001, fMLF or WKYMVM diluted in KRG buffer supplemented with 0.3% BSA, were added to wells in the lower chamber. Cell suspensions (30 µl) containing neutrophils (2 × 10^6^/ml, isolated from peripheral blood) were placed on top of the filter and allowed to migrate for 90 min at 37°C. The cell migration to the bottom well was visualized under microscope and for quantitative analysis the content of MPO (a marker protein present in the neutrophil azurophil granules) was assessed in the lysates (cells in lower chamber treated with 2% BSA and 2% CTAB for 60 min, at room temperature) by addition of OPD and hydrogen peroxide. Random neutrophil migration (no attractant present) was also determined and the recruitment was expressed as the enzyme activity (absorbance unit of optical density at 450 nm, AU) of the cells recovered from the bottom wells after migration.

### 2.8 β‐arrestin 2 recruitment assay

The ability of agonists in promoting FPRs to β‐arrestin was evaluated in the PathHunter eXpress Chinese hamster ovary (CHO) cells‐K1 FPR1 or FPR2 cells from DiscoverX (Fremont, CA, USA) which co‐express ProLink tagged FPR1 or FPR2 and an enzyme acceptor tagged β‐arrestin so that β‐arrestin binding can be measured via enzyme fragment complementation as increased β‐galactosidase activity. The assay was performed according to manufacturer's instructions as previously described.^30^, ^34^ In brief, cells were seeded in tissue culture treated 96‐well plates (10^4^ cells/well) and incubated at 37°C, 5% CO_2_ for 20 h. The cells were then incubated with agonists (90 min, 37°C), followed by addition of detection solution and incubated for another 60 min at room temperature. The CL was measured on a CLARIOstar plate reader (BMG Labtech, Ortenberg, Germany).

### 2.9 Phosphorylation of ERK1/2 determined by electrochemiluminescence

Human neutrophils (2 × 10^6^/ml) were stimulated with fMLF or RE‐04‐001 for 2 min followed by rapidly cool down to stop reaction with ice old lysis buffer provided by Meso Scale Diagnostics (MSD, Rockville, MD, USA) according to manufacturer's instructions as described.^31^ The lysis was performed on ice for at least 30 min and the supernatant was collected and stored at −80°C before use. Phosphorylation of ERK1/2 (pERK) was measured using the MSD electrochemiluminescence technology with phospho(Thr202/Tyr204; Thr185/Tyr187)/Total ERK1/2 assay whole lysate kit (Cat#K151DWD) according to manufacturer's instructions. Data are presented as % pERK = ([2× phospho‐signal]/[phospho‐signal + total signal]) × 100.

### 2.10 Data analysis

Data analysis was performed using GraphPad Prism 8.0 (Graphpad Software, San Diego, CA, USA). Curve fitting was performed by nonlinear regression using the sigmoidal dose–response equation (variable slope). Statistical analysis was performed on raw data values using either a repeated measurement 1‐way ANOVA followed by Dunnett's multiple comparison post‐hoc test or a paired Student's *t*‐test. Statistically significant differences are indicated by *P < 0.05 and **P < 0.01.

## 3 RESULTS

### 3.1 RE‐04‐001 activates human neutrophils

**FIGURE 1:**
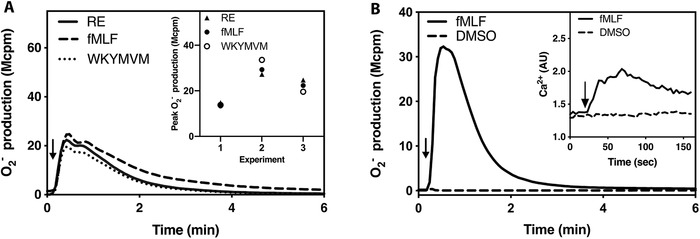
**The novel small compound RE‐04‐001 (for short—RE—in figures and legends) activates neutrophil‐like HL60 cells. (A)** A sensitive technique to measure superoxide production was used to determine the ability of RE to activate neutrophil‐like HL60 cells (10^5^ cells). Cells were pre‐incubated at 37°C for 5 min before agonist stimulation (indicated by arrows) with RE (100 nM, solid line), the formyl peptide receptor (FPR)1 agonist fMLF (100 nM, dashed line) and the FPR2 agonist WKYMVM (100 nM, dotted line). A representative experiment out of three independent experiments is shown. **Inset**: The peak O_2_^−^ production induced by RE (triangles) and the two established FPR agonists fMLF (closed circles) and WKYMVM (open circles) from three independent experiments are shown. (**B)** The activity of DMSO (vehicle control, dashed lines) on its own in activating neutrophil‐like HL60 cells was measured by its ability to trigger a release of superoxide. **Inset**: a transient rise in intracellular Ca^2+^ ([Ca^2+^]_i_). As a positive control, fMLF (100 nM and 10 nM, respectively) was used in parallel to trigger the oxidase release and a transient rise in [Ca^2+^]_i_ (solid lines)

A compound library containing drug‐like small molecules was used in a screening study to identify novel NADPH‐oxidase activators.^33^ The release of O_2_^−^ from neutrophil‐like HL60 cells was determined, and RE‐04‐001 was found to activate the NADPH‐oxidase (Fig. 1). In addition as secondary pharmacology screen, the activity of this compound (≥50% inhibition or stimulation) was determined using the HitProfilingScreen provided by Eurofins, which includes a panel of 30 common drug targets. No activating or inhibitory effects were induced by RE‐04‐001 at any of the targets determined with concentrations of the compound up to 10 µM (Supporting Information Table S1). It is clear from the results obtained, that the activation pattern of RE‐04‐001 was similar in magnitude and time course, to that induced by the two high‐affinity FPR agonists fMLF (specific for FPR1) and WKYMVM (specific for FPR2) (Fig. 1A). As a negative control, vehicle control DMSO alone induced neither a release of the superoxide nor a transient rise in the intracellular concentration of free calcium ions ([Ca^2+^]_i_), whereas both assay systems could be activated by fMLF (Fig. 1B).

**FIGURE 2:**
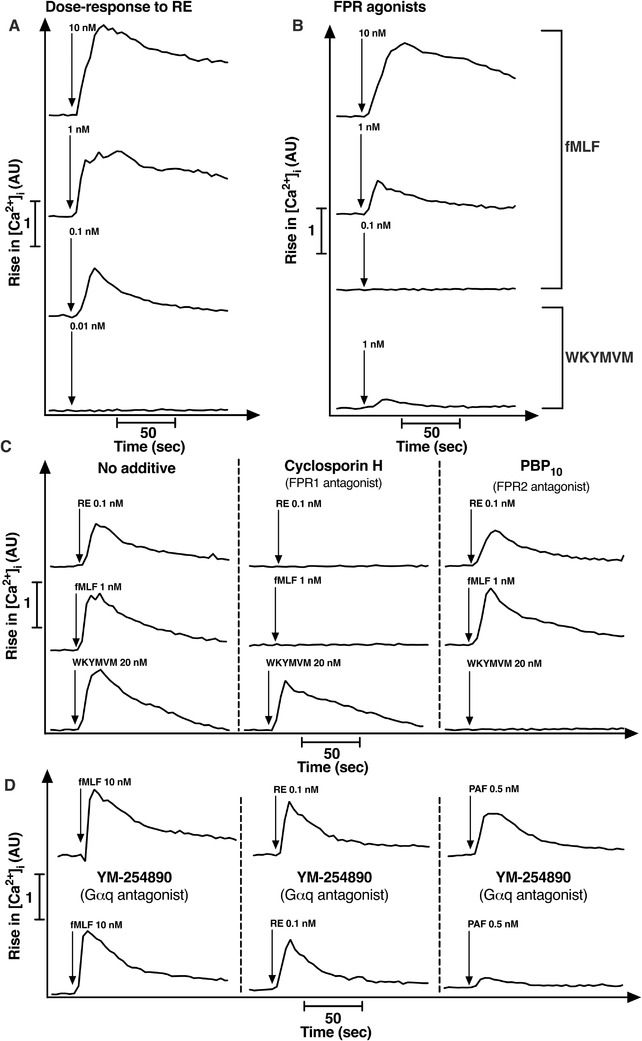
**The small compound RE triggers formyl peptide receptor (FPR)1‐mediated intracellular rise of Ca^2+^ independent of Gαq protein activation in human neutrophils**. Prior reports show that FPRs primarily use a Gαi rather that a Gαq containing G protein to induce a transient rise of intracellular Ca^2+^ ([Ca^2+^]_i_) in human neutrophils. To investigate the RE receptor preference and G protein coupling the transient rise in [Ca^2+^]_i_ was determined in human neutrophils. **(A)–(B)** The transient rise of [Ca^2+^]_i_ in neutrophils was induced by different concentrations of RE (10 nM to 0.01 nM), fMLF (10 to 0.1 nM) and WKYMVM (1 nM). (**C)** Effect of the FPR1 antagonist cyclosporin H (CysH, 1 µM, middle panel) or the FPR2 antagonist PBP_10_ (1 µM, right panel) on the transient rise of [Ca^2+^]_i_ induced by RE (0.1 nM), fMLF (1 nM), and WKYMVM (20 nM). Control cells received no antagonist (left panel). Agonist addition was indicated by arrows. (**D)** Neutrophils received YM‐254890 (a selective Gαq inhibitor; 200 nM) 5 min before stimulation with fMLF (10 nM), RE (0.1 nM), or PAF (0.5 nM). Control cells received no YM‐254890. (**A)–(D)** Representative traces from three independent experiments are shown

It is well known that both FPR1 and FPR2 are abundantly expressed by human neutrophils and the receptors recognize numerous structurally unrelated agonists.^3^, ^9^ The similarity in the responses both in kinetics and in magnitude, induced by RE‐04‐001 and the two FPR peptide agonists (Fig. 1A) suggested that RE‐04‐001 could be an FPR agonist that should activate also primary blood neutrophils. One of the very early signaling events downstream of activated neutrophil FPRs is a transient increase in [Ca^2+^]_i_, an event initiated by a G‐protein‐dependent activation of phospholipase C and a release of Ca^2+^ from intracellular storage organelles.^38^ Hence, we could show that RE‐04‐001 induced a robust and concentration‐dependent rise in [Ca^2+^]_i_ in human neutrophils (Fig. 2A). RE‐04‐001 induced a full response already at a 1 nM concentration, and the activity was retained even at concentrations down to 0.1 nM, a concentration not able to trigger a rise in [Ca^2+^]_i_ with either fMLF or WKYMVM (Fig. 2B). Also, in the Ca^2+^ assay system using human neutrophils, RE‐04‐001 and the FPR peptide agonists triggered very similar response, further suggesting that RE‐04‐001 may interact with FPRs to mediate its biologic responses in neutrophils. To determine the involvement of FPRs in the RE‐04‐001‐induced neutrophil activation, we used two well‐known receptor‐specific antagonists, cyclosporine H (antagonizes primarily FPR1; Stenfeldt et al.^39^) and PBP10 (antagonizes primarily FPR2; Berridge^40^). The results obtained with these antagonists clearly show that RE‐04‐001 is recognized by FPR1 (Fig. 2C). Based on the lack of inhibition with the FPR2 antagonist, we conclude that FPR2 is not of importance for the RE‐04‐001‐induced activity (Fig. 2C). For comparison, control experiments with prototype peptide agonists for FPR1 and FPR2 are included to show that cyclosporine H selectively inhibits the fMLF‐induced response, whereas PBP10 inhibits the WKYMVM response without any effect on the fMLF‐induced response (Fig. 2C).

For many GPCRs, the transient rise in [Ca^2+^]_i_ upon agonist binding is achieved through an activation of a Gαq containing G protein followed by the activation of downstream PLC‐PIP_2_‐IP_3_ pathway leading to the emptying of [Ca^2+^]_I_ stores.^41^, ^42^ One such Gαq‐linked neutrophil receptor is the platelet‐activating factor receptor (PAFR),^43^ and accordingly, the PAF‐induced rise in [Ca^2+^]_i_ was inhibited by the selective Gαq inhibitor YM‐254890 (Fig. 2D). In contrast to the PAFR, the FPR‐mediated [Ca^2+^]_i_ response does not engage Gαq but the heterodimeric Gβγ subunit derived from a Gαi containing G protein.^43^ The fact that the rise in [Ca^2+^]_i_ induced by RE‐04‐001 was insensitive to the Gαq selective inhibitor (Fig. 2D), is in line with the notion that RE‐04‐001 interacts with FPR1 and that the [Ca^2+^]_i_ rise is achieved through a Gαi containing G protein. Taken together, these data clearly show that RE‐04‐001 activates human neutrophils manifested as a rise in [Ca^2+^]_i_, and the response is sensitive to an antagonist of FPR1—but not to one for FPR2—or to a Gαq selective inhibitor.

### 3.2 RE‐04‐001 activates neutrophils to release O_2_^−^

**FIGURE 3:**
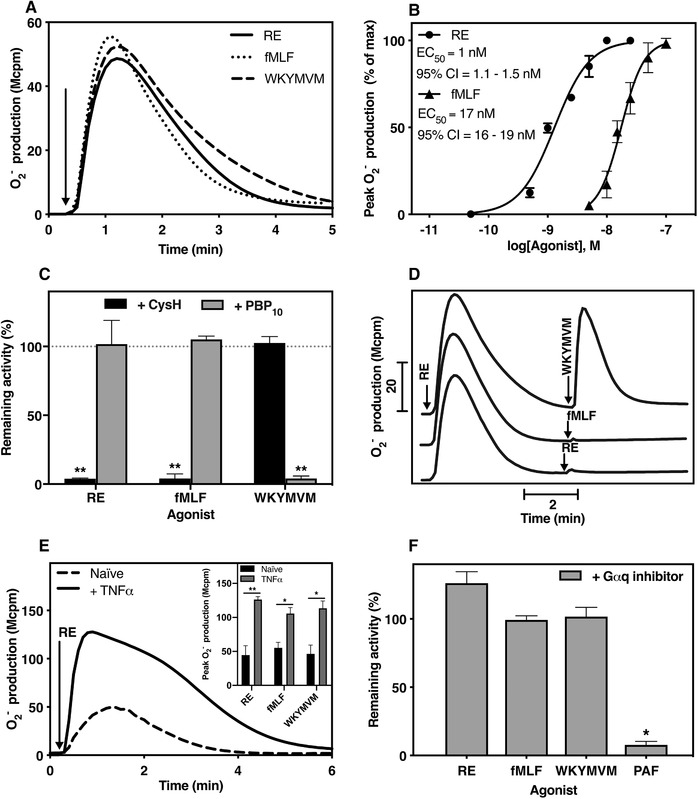
**The small compound RE induces formyl peptide receptor (FPR)1‐mediated NADPH‐oxidase activation independent of Gαq protein activation from human neutrophils**. Prior reports show that FPR1 agonist activate the neutrophil NADPH‐oxidase and the response involves a Gαi containing G protein. To investigate the RE receptor preference and G protein coupling the release of superoxide anions (O_2_^−^) was determined in human neutrophils. **(A)** Neutrophils were stimulated with RE (10 nM, solid line), fMLF (100 nM, dotted line), or WKYMVM (100 nM, dashed line). One representative trace of O_2_^−^ production out of three independent experiments is shown. (**B)** Dose–response of RE and fMLF. The EC_50_‐values and 95% confidence interval (CI) were determined based on the peak O_2_^−^ response (*n* = 3). (**C)** Effect of CysH (1 µM, black bars) or PBP_10_ (1 µM, gray bars) pre‐incubated with neutrophils for 5 min before activation with RE (10 nM), fMLF (100 nM), or WKYMVM (100 nM). The data are presented as percent of remaining NADPH‐oxidase activity in the presence of antagonists as compared to the responses from control cells (mean ± sd, *n* = 3). One‐way ANOVA followed by Dunnett's post‐hoc test was used to calculate significance. (**D)** Neutrophils were first stimulated with RE‐04‐001 (10 nM, arrow to the left) and then further challenged a second stimulation with WKYMVM (100 nM), fMLF (100 nM), or RE (10 nM) as indicated. (**E)** Naïve or TNFα (37°C, 20 min) primed neutrophils were challenged with RE (10 nM). One representative trace out of three independent experiments is shown. **Inset**: Comparison between the peak O_2_^−^ responses released from naïve (black bars) and TNFα primed cells (gray bars) stimulated with RE (10 nM), fMLF (100 nM), or WKYMVM (100 nM). Data are presented as mean ± sd (*n* = 3) and paired *t*‐test was used to calculate the TNFα priming effect. **(F)** Comparison between the peak O_2_^−^ responses released by neutrophils pretreated with or without the YM‐254890 (200 nM) for 5 min before activation with RE (10 nM), fMLF (100 nM), WKYMVM (100 nM), or PAF (100 nM). Data are presented as percent of remaining NADPH‐oxidase activity in the presence of YM‐254890, compared to the responses from control cells (mean ± sd, *n* = 3). Paired *t*‐test was used to calculate the effect of YM‐254890

To further assess neutrophil activation by RE‐04‐001, we determined the ability of the compound to trigger an assembly of the O_2_^−^ generating NADPH‐oxidase in human neutrophils. We show that RE‐04‐001 activates neutrophils to release O_2_^−^, and there was a very rapid onset of the response that was then terminated in around 5 min after the initiation, a response pattern very similar to that induced by the two prototype FPR peptide agonists (Fig. 3A). The maximal level of O_2_^−^ production induced by RE‐04‐001 was of the same magnitude as that induced by 100 nM fMLF, suggesting that RE‐04‐001 is a full agonist (Fig. 3A). The response induced by RE‐04‐001 was concentration dependent with an EC_50_ value in the low nanomolar range (Fig. 3B), which is much lower than that for the prototype FPR1 agonist fMLF (EC_50_ ≈ 20 nM; Fig. 3B). In line with the data obtained with FPR‐specific antagonists in the [Ca^2+^]_i_ assay system (Fig. 2C), the inhibitory profile for RE‐04‐001 was the same as that that of fMLF (sensitive to cyclosporine H but not to PBP_10_) but different from WKYMVM (Fig. 3C).

The preference of RE‐04‐001 for FPR1 over FPR2 in human neutrophils gained further support from receptor homologous desensitization experiments. Neutrophils first activated with RE‐04‐001 were not only homologously desensitized (nonresponsive) to a second stimulation with RE‐04‐001 but were also refractory to stimulation with fMLF (Fig. 3D). In contrast, these RE‐04‐001 desensitized cells were still fully responsive to a second stimulation with the FPR2 agonist WKYMVM (Fig. 3D). Taken together, these data show that RE‐04‐001 is a very potent stimulus that activates the neutrophil NADPH‐oxidase and this activation is achieved through signals specifically generated by FPR1.

It is well known that the NADPH‐oxidase activity triggered by FPR‐specific agonists is substantially increased in TNFα primed neutrophils.^34^, ^44^ Accordingly, the amount of O_2_^−^ produced by TNFα primed cells was substantially increased with RE‐04‐001 as the activating FPR agonist (Fig. 3E). The increase due to priming with TNFα was of the same magnitude as that with fMLF and WKYMVM (Fig. 3E inset). Finally, in agreement with the lack of inhibitory effect of the Gαq inhibitor on the FPR‐mediated rise in [Ca^2+^]_i_ (Fig. 2D), the O_2_^−^ production induced by RE‐04‐001 and other FPR agonists (i.e., peptides fMLF and WKYMVM) was not inhibited by the Gαq inhibitor YM‐254890 (Fig. 3F). The inhibitory effects of the Gαq inhibitor on PAF‐induced NADPH‐oxidase activity is shown for comparison (Fig. 3F). Taken together, these data show that RE‐04‐001 is a potent and full agonist selective for FPR1, and the agonist activates the neutrophil NADPH‐oxidase independent of coupling to a Gαq containing G protein.

### 3.3 Comparison of neutrophil chemotaxis induced by fMLF and RE‐04‐001

**FIGURE 4:**
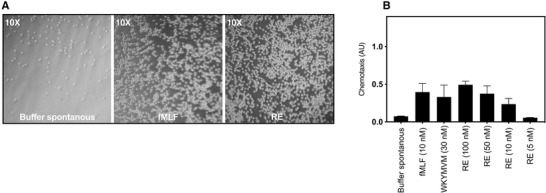
**Neutrophil chemotaxis induced by fMLF and RE**. Neutrophil migration toward RE and fMLF placed in the bottom wells was determined. (**A**) Representative micrographs (10× magnification) of neutrophils migrated into the lower compartment containing buffer (spontaneous migration), fMLF (10 nM), or RE (50 nM). (**B)** Quantification of neutrophil migration toward fMLF (10 nM), WKYMVM (30 nM), or different concentrations of RE by analyzing the amount of myeloperoxidase (MPO) in cells recovered from the lower compartments after 90 min of migration period. Data are presented as chemotaxis index (AU, MPO activity of cells recovered from the bottom wells after migration in absorbance unit at 450 nm) from three independent experiments (mean + sd, *n* = 3)

Based on the fact that the prototype FPR1 agonist fMLF and a large number of other earlier described FPR agonists potently recruit neutrophils, the FPRs are termed chemoattractant receptors.^3^, ^4^ This generalization is, however, not completely valid, as shown by the results obtained with some FPR2 agonists such as lipidated peptides (pepducins and peptidomimetics) and the formylated peptides belonging to the group of phenol soluble modulins (PSMα peptides). Despite being potent activators in promoting superoxide release, these agonists lack completely the ability to induce neutrophil chemotactic migration.^29^, ^30^, ^31^ To determine the chemotactic activity of RE‐04‐001, we used the transwell chamber system in which neutrophils (placed in the upper chamber) were allowed to migrate through a filter that separates the agonist (placed in the bottom well in the chambers) from the cells. The FPR1 peptide agonist fMLF was used as positive control (Fig. 4A), and in accordance with the common relation between the attraction concentrations needed to induce chemotaxis and activate the NADPH‐oxidase, respectively, lower concentrations of fMLF were needed to trigger chemotaxis. RE‐04‐001 attracted neutrophils to a level similar to that induced by fMLF (Fig. 4B), but comparably higher concentrations of RE‐04‐001 were required (Fig. 4B), suggesting that signaling downstream RE‐04‐001 activated FPR1 is functional selective, that is, in favor of oxidase activation over chemotaxis. To directly compare the functional selective profile of RE‐04‐001, biased signaling ratios were calculated; ratios of 0.1 and 5 were obtained for RE‐04‐001 and fMLF, respectively. These values were calculated by a direct comparison of the respective EC_50_ value for activation of the NADPH‐oxidase with that to recruit neutrophils chemotactically. Although the migration induced by a 50 nM concentration of RE‐04‐001 reached the same level as that obtained with the optimal concentration of fMLF, the differences between the two agonist in the functional selective ratio values, clearly show that RE‐04‐001 induced a functional selective response, being biased toward ROS production (Fig. 3B) and away from chemotaxis (Fig. 4B).

### 3.4 RE‐04‐001 activates ERK1/2 phosphorylation rather than promoting β‐arrestin recruitment

**FIGURE 5:**
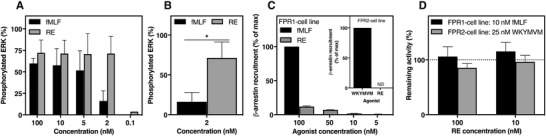
**The small compound RE potently triggers ERK1/2 phosphorylation but poorly recruits β‐arrestin. (A)** Phosphorylation of ERK1/2 (pERK1/2) was determined in neutrophil lysates after stimulation with different concentrations of fMLF or RE as indicated for 2 min. Data are presented as percentage of phosphorylated ERK (% pERK) from two to three independent experiments that were run with duplicates (mean + sd). (**B)** Comparison of phosphorylated ERK induced by fMLF (2 nM) and RE (2 nM) and Student's paired *t*‐test was used to calculate statistics. *P < 0.05 (**C)** β‐arrestin recruitment was monitored in CHO cells over‐expressing FPR1 stimulated with 100 nM of fMLF or different concentrations of RE as indicated. Data are presented as percentage of the response induced by 100 nM fMLF (mean + sd, *n* = 3). **Inset**: FPR2 over‐expressing CHO cells were stimulated with the FPR2 agonist WKYMVM (100 nM) or RE (100 nM). Data are presented as percentage of the response induced by 100 nM WKYMVM. (**D)**. Effect of RE on the fMLF response (10 nM) and WKYMVM response (25 nM) in FPR1 cells (black bars) and FPR2 cells (gray bars), respectively. Data are presented as percentage of remaining β‐arrestin recruitment in the presence of RE‐04‐001 as compared to the responses from control cells (mean + sd, *n* = 3)

The functional selectivity profile of RE‐04‐001 in human neutrophils suggests that the agonist triggers a biased signal cascade downstream FPR1. In addition to a rise in [Ca^2+^]_i_, many FPR agonists trigger also ERK1/2 phosphorylation and recruitment of cytosolic β‐arrestin to cytoplasmic parts of the activated receptors.^45^ For many GPCRs the latter event is of importance for receptor desensitization and internalization as well as for the transduction of noncanonical signals of which activation of ERK1/2 may be one.^46^ Phosphorylation of ERK1/2 in human neutrophils upon agonist stimulation was determined as previously described.^31^ Similar to potent agonistic activity by RE‐04‐001 in inducing a rise in [Ca^2+^]_i_, the agonist induced also ERK1/2 phosphorylation and the potency was comparable to, or slightly higher than that of fMLF (Fig. 5A). The ability of RE‐04‐001 to promote receptor‐mediated recruitment of β‐arrestin was studied in CHO cells overexpressing FPR1.^30^ In contrast to the potent activity of RE‐04‐001 in inducing a transient rise in [Ca^2+^]_i_ and ERK1/2 phosphorylation (Fig. 2A, 5A), the amount of β‐arrestin recruited by RE‐04‐001 in FPR1 overexpressing cells was negligible in comparison to that induced by fMLF (Fig. 5B). In agreement with the receptor specificity of RE‐04‐001, this agonist did not recruit any β‐arrestin in FPR2 overexpressing cells; the FPR2 agonist WKYMVM was included as an FPR2 control for comparison (Fig. 5B inset).

When comparing β‐arrestin recruitment induced by the FPR1 agonist fMLF and RE‐04‐001, respectively, it is clear that whereas a full recruitment is achieved with a 10 nM concentration of fMLF, a very low level of β‐arrestin recruitment (less than 20%) was obtained with much higher RE‐04‐001 concentrations (Fig. 5B). Despite the fact that RE‐04‐001 is potent FPR1 agonist determined as a transient rise in [Ca^2+^]_i_, ERK1/2 phosphorylation and, activation of the NADPH‐oxidase, RE‐04‐001 did not block fMLF‐induced β‐arrestin recruitment even when the RE‐04‐001 concentration was 10 times (100 nM) that of fMLF (Fig. 5C). As expected, RE‐04‐001 lacked an effect also on FPR2 agonist WKYMVM‐induced β‐arrestin recruitment (Fig. 5C). The functional selective profile of RE‐04‐001, away from chemotaxis and β‐arrestin recruitment in comparison to its potent activity for ROS release and a transient rise in [Ca^2+^]_i_ as well as ERK1/2 phosphorylation, is in line with our earlier signaling profile of functional selective FPR2 agonists.^29^, ^30^, ^31^

Taken together, these data clearly show that the FPR1 agonist RE‐04‐001 displays not only a functional selectivity (NADPH‐oxidase over chemotaxis) but also a strong signaling bias in favor of the signal giving rise to an increase in [Ca^2+^]_i_ and ERK1/2 phosphorylation over that resulting in β‐arrestin recruitment.

### 3.5 RE‐04‐001 promotes FPR1 to crosstalk with other neutrophil receptors

**FIGURE 6:**
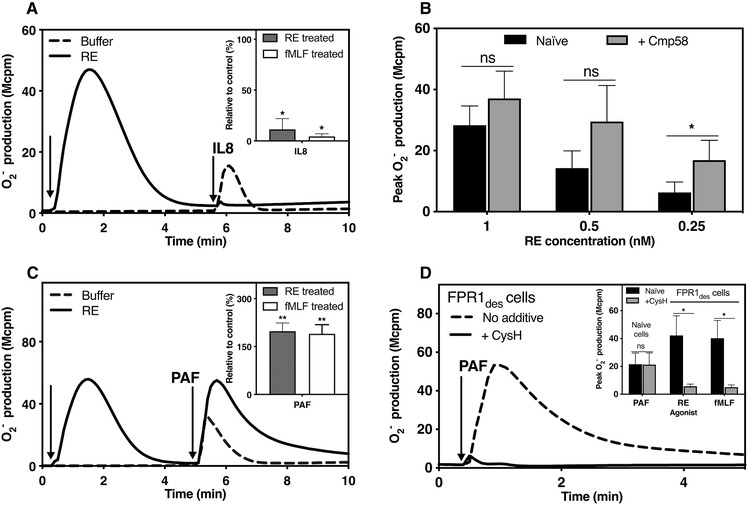
**The small compound RE activated FPR1 modulates other GPCR‐mediated neutrophil response**. Receptor crosstalk was studied in the NADPH‐oxidase activation assay by measuring the superoxide anions (O_2_^−^) production from cells desensitized with RE‐04‐001 and from naïve cells received no RE‐04‐001. (**A)** Crosstalk between RE activated FPR1 and IL8. Neutrophils were activated with RE‐04‐001 (10 nM, indicated by the first arrow) when the response had declined, the cells received a second dose of IL8 (100 ng/ml; indicated by the second arrow). **Inset**: Quantification of the second IL8 response in RE or fMLF pre‐activated cells from three independent experiments (mean + sd, *n* = 3). (**B)** A second dose of different concentrations of RE was added to cells pretreated with Cmp58 (1 µM), the FFAR2 allosteric modulator. Peak O_2_^−^ production are shown from three independent experiments (mean + sd, *n* = 3). (**C)** RE (10 nM, indicated by the first arrow) activated cells received a second stimulation with PAF (100 nM; indicated by the second arrow). One representative experiment out of three independent experiments is shown. **Inset**: Quantification of the second PAF‐response from neutrophils prestimulated with either RE (10 nM) or fMLF (100 nM). Data are presented as percentage of control response (not pre‐activated) (mean + sd, *n* = 4). (**D)** Neutrophils were desensitized with RE (10 nM) to obtain FPR1_des_ cells before a second stimulation with PAF (100 nM). The FPR1 antagonist CysH was added just prior PAF stimulation (solid line) or cells received no addition before PAF stimulation (dashed line). Representative traces of O_2_^−^ production is shown. **Inset**: Peak O_2_^−^ production induced by PAF from naïve cells and FPR_des_ cells desensitized with RE or fMLF (100 nM) received with or without CysH are shown (mean + sd, *n* = 4). Paired Student's *t*‐test was used to calculate statistical significance between treated and control groups

Following the response induced in neutrophils challenged with the FPR1 agonist fMLF or RE‐04‐001, the receptors/cells are transferred to a homologous desensitized state in which the cells are nonresponsive to second agonist dose (Fig. 3D). There is a known hierarchy between different neutrophil GPCRs, and in this hierarchy FPR1 is ranked higher than the receptors for the cytokine IL‐8 regarding both the NADPH‐oxidase activation and neutrophil chemotaxis.^47^, ^48^ In accordance with this, the homologous FPR1 desensitization induced by fMLF and RE‐04‐001 was accompanied by a concomitant inhibition (heterologous desensitization) of the response mediated by the IL‐8 receptors, making these FPR1 desensitized cells nonresponsive to IL‐8 stimulation (Fig. 6A).

Recent research suggests that the receptor crosstalk hierarchy is complex and not only desensitized receptors but also allosteric modulated GPCRs can communicate with other receptors.^45^, ^49^, ^50^ A prominent example of such a crosstalk is that FPRs signaling can be positively regulated by free fatty acid receptor 2 (FFAR2) as illustrated by the fact that neutrophils with their FFARs allosterically modulated are primed when activated by low (normally nonactivating) concentrations of FPR agonists.^51^, ^52^ The fact that RE‐04‐001 response induced in neutrophils in the presence of an allosteric FFAR2 modulator, is inhibited not only to an FPR1 antagonist but also by an antagonist specific for FFAR2 (Fig. 6B) shows that this response is achieved through receptor crosstalk between FPR1 and FFAR2.

Opposite to the heterologous inhibitory effect of RE‐04‐001 on the IL‐8 response (Fig. 6A), a substantially enhanced PAF response was induced in FPR1‐desensitized neutrophils compared to the PAF response from control cells not receiving any RE‐04‐001 (Fig. 6C) and no difference was observed in cells when desensitized by fMLF or RE‐04‐001 (Fig. 6C inset). The involvement of FPR1 in this response is evident from the fact that the second PAF response in RE‐04‐001 desensitized cells is sensitive to the FPR1 antagonist cyclosporine H when added just prior to PAF stimulation (Fig. 6D). This is in line with the earlier data showing that PAF/PAFR is able to transduce a not yet known signal leading to a reactivation of neutrophils with desensitized FPRs.^53^

In summary, we show that the novel FPR1 agonist RE‐04‐001, despite its biased signaling feature, similar to fMLF places FPR1 in the same position in the neutrophil receptor hierarchy and allows receptor crosstalk with other GPCRs to either suppress or amplify the neutrophil response.

### 3.6 The termination of the RE‐04‐001‐induced activation of the NADPH‐oxidase is regulated primarily by the actin cytoskeleton rather than by β‐arrestin

**FIGURE 7:**
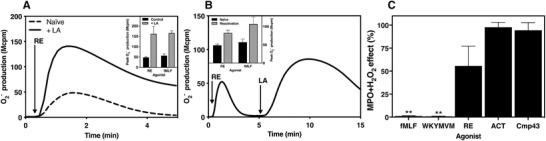
**Modulation of the small compound RE activity by latrunculin A and the myeloperoxidase (MPO)/H_2_O_2_ system**. The NADPH‐oxidase activity of neutrophils was determined. (**A)** Naïve neutrophils and neutrophils pre‐incubated with the actin cytoskeleton‐disrupting drug latrunculin A (LA; 25 ng/ml, 5 min) were activated with RE (10 nM). One representative experiment of three independent experiments is shown. **Inset**: The peak NADPH‐oxidase activities induced in naïve or LA treated neutrophils by RE (10 nM) or fMLF (100 nM) are shown (mean + sd, *n* = 3). (**B)**. Neutrophils activated by RE (10 nM, first arrow), when the response had declined, were reactivated with LA (25 ng/ml, second arrow). One representative experiment of three independent experiments is shown. **Inset**: The peak NADPH‐oxidase activity induced by RE (100 nM), or fMLF (100 nM) from naïve neutrophils and that during reactivation with LA are shown (mean + sd, *n* = 3). (**C)**. Oxidization of the agonist toward the MPO (1 µg/ml) + H_2_O_2_ (10 µM) system. The remaining activity of agonist after oxidization was measured by their ability to trigger ROS production from neutrophils in comparison to the control response with agonists received no MPO‐H_2_O_2_ from three independent experiments (mean + sd, *n* = 3). The final concentrations of agonists used in the oxidase assay: fMLF (100 nM); WKYMVM (100 nM); RE (12.5 nM); Act‐389949 (ACT, 12.5 nM); and Cmp43 (250 nM). Paired *t*‐test was used to calculate the statistical significance of agonist treated with or without MPO‐H_2_O_2_

Despite the fact that β‐arrestin plays an important role in receptor desensitization for many GPCRs, we and others have demonstrated that the actin cytoskeleton, rather than the recruited β‐arrestin, constitutes the basis for FPR desensitization and termination of signals that activate the ROS‐producing oxidase.^45^, ^54^, ^55^ This notion gains further support from the fact that FPR1 is homologously desensitized also by the non‐β‐arrestin recruiting agonist RE‐04‐001. In addition, in neutrophils pretreated with the actin cytoskeleton disrupting agent latrunculin A, RE‐04‐001‐induced activation resulted in a 4‐fold higher superoxide production in comparison to that produced by the cell in the absence of latrunculin A (Fig. 7A). Furthermore, RE‐04‐001 activated neutrophils transferred to a nonsignaling desensitized state were resensitized/reactivated and produce ROS when the actin cytoskeleton was disrupted through the addition of latrunculin A (Fig. 7B). These data, obtained with RE‐04‐001 as an activating agonist, are in agreement with the pattern induced by fMLF (Fig. 7A and B). Taken together, we show that RE‐04‐001‐induced FPR1 desensitization in neutrophils occurs primarily through the involvement of an intact actin cytoskeleton.

### 3.7 RE‐04‐001 is resistant to oxidation by the MPO‐H_2_O_2_‐halide system

Processing of NADPH‐oxidase‐derived hydrogen peroxide (H_2_O_2_) by MPO, a neutrophil enzyme stored in the azurophil granules, results in a generation of highly reactive oxidants that regulate many biologic processes in addition to bacterial killing.^15^, ^37^, ^56^ In line with this, we have earlier shown that the MPO‐H_2_O_2_‐halide system inactivates peptide agonists such as the FPR1 selective fMLF and the FPR2 selective WKYMVM.^34^, ^57^ This inactivation is evident from the inability of the oxidized peptides to activate and trigger ROS release from neutrophils. In contrast to the two peptide agonists, the two small compound agonists Cmp43 (dual FPR agonist) and Act‐389949 (FPR2 selective) are completely resistant to the MPO‐H_2_O_2_‐halide radical system.^34^, ^58^ To study the inhibitory effect on RE‐04‐001 of the MPO‐H_2_O_2_‐halide system, the agonist was incubated with the MPO+H_2_O_2_ system, and the ability of the ROS‐treated compounds to activate the neutrophils NADPH‐oxidase system was determined and compared to activity induced by the non‐ROS treated agonists. For comparison, the effects of the MPO+H_2_O_2_ system on four control compounds were investigated in parallel. As expected, no NADPH‐oxidase activity was induced by the MPO+H_2_O_2_ treated control peptides fMLF or WKYMVM (Fig. 7C), the peptides earlier shown to be ROS sensitive.^34^, ^57^ No inhibition was seen when Cmp43 and Act‐389949 were exposed to the MPO‐H_2_O_2_‐halide generating system (Fig. 7) results also in agreement with earlier findings.^34^, ^58^ Compared to the FPR1 selective peptide agonist fMLF, RE‐04‐001 was fairly resistant to the MPO‐H_2_O_2_‐halide radical system (Fig. 7C). Taken together, these data show that the RE‐04‐001 resists inactivation induced by the MPO‐H_2_O_2_‐halide system.

## 4 DISCUSSION

In this study, we show that the small molecule RE‐04‐001 activates human neutrophils and the agonist is specifically recognized by FPR1, one of the pattern‐recognition FPRs. In‐depth characterization of the FPR1 agonist reveal that there are striking similarities between RE‐04‐001 and the prototype peptide agonist fMLF, with the exception that signaling by RE‐04‐001 is biased in favor of the signals giving rise to an increase in [Ca^2+^]_i_ and ERK1/2 phosphorylation over those recruiting β‐arrestin, a signaling profile linked to a functional selective (NADPH‐oxidase over chemotaxis) neutrophil response.

Peptides with a formylated methionine in their N‐terminus, a hallmark of protein/peptide synthesized by bacteria and mitochondria, are recognized by the innate immune system through high affinity binding of formylated peptides to FPR1 and/or FPR2, receptors expressed primarily in myeloid cells such as granulocytes and monocytes/macrophages.^3^, ^4^, ^45^ Following early work showing that formylated peptides are high affinity FPR ligands, FPR1 as well as the closely related FPR2 have been shown to be promiscuous and recognize also a large number of compounds lacking the formylated methionine. Some of the potent FPR2 agonists have been shown to be functional selective, but very few of the FPR1 selective agonists displaying biased signaling and functional selective properties have been described. At present, only a few FPR agonists have progressed to clinical trials. For example, the dual FPR1/2 agonist compound 17b has been reported to exert anti‐inflammatory effects and protect mice from myocardial infarction injury,^59^ whereas another compound (Bristol Meyers‐Squibb; BMS‐986235) has proceeded into a clinical phase I study as a selective FPR2 agonist for prevention of heart failure.^60^ Yet another FPR2 selective agonist Act‐389949 entered a clinical phase I study but the data obtained show that the surface exposed neutrophil receptors were rapidly lost, although the mechanisms for this were not described.^61^ It is clear that better understanding of the basic biology and of the mechanisms that regulate FPRs is highly desirable, and such in‐depth analyses should preferentially include a determination of the precise roles of FPR1 and FPR2, the receptor desensitization and receptor crosstalk profiles as well as the intracellular signals generated (including recruitment of β‐arrestin), together with the downstream cellular response and therapeutic effects. In line with this, we have attempted to characterize the effect of RE‐04‐001 on the main FPR1 downstream signaling pathways, and these include ERK1/2 phosphorylation, the PLC‐PIP_2_‐IP_3_‐dependent change in the cytosolic concentration of Ca^2+^, and recruitment of β‐arrestin.

From the results presented, we conclude that the neutrophil‐activating agonist RE‐04‐001 is recognized by FPR1 and this conclusion is primarily based on the following findings: (i) the RE‐04‐001‐induced activation of neutrophil is inhibited by the earlier well‐characterized FPR1 antagonist cyclosporin H but not by the FPR2‐specific antagonist PBP_10_ ^39^; (ii) RE‐04‐001 homologously desensitizes neutrophils to the FPR1 agonist fMLF but not to the FPR2 agonist WKYMVM and the same type of selective desensitization is obtained when fMLF is used as the desensitizing agonist; and (iii) RE‐01‐004 at high concentrations induced a small recruitment of β arrestin in cells expressing FPR1 but no such recruitment is achieved in cells expressing FPR2. The promiscuous ligand binding feature for the FPRs^3^, ^4^ now includes also RE‐04‐001, but the precise structural requirements for ligand recognition by the FPRs are still poorly understood. Two very recent structure biology studies have, however, revealed the crystal structure of FPR2 in its active conformation in complex with the high affinity peptide agonist WKYMVm.^62^, ^63^ Future molecular docking of FPR1 selective agonists using an FPR2‐based model of FPR1 may define the mechanistic insights into FPR1 selective recognition of such compounds. These types of studies may also reveal differences in conformational and binding modes between fMLF and RE‐04‐001. The fact that RE‐04‐001 lacks the ability to block the β‐arrestin recruitment induced by fMLF suggest that the two FPR1 agonists binds to different sites on the receptor that agonist dependently transduce distinct signaling pathways and trigger different cellular responses (see discussion in the following text).

It is generally accepted that activation by receptor specific agonists of chemoattractant GPCRs such as the FPRs, regulates the recruitment of neutrophils from the blood stream to inflammatory sites in infected/damaged tissues and the receptors’ downstream signals induce the release/secretion of proteolytic enzymes and ROS.^3^ We show that similar to the FPR1 agonist fMLF, RE‐04‐001 acts as a full agonist for activation of the ROS generating NADPH‐oxidase, and the level of ROS production is largely amplified/primed in cells pretreated with TNFα. The precise molecular background to the TNFα primed response is not known but it may be the result of an increased exposure of membrane receptors mobilized from stores in the secretory granules. Our earlier studies have demonstrated that such secretory organelles containing CD11b, FPR1, and FPR2 are mobilized to the neutrophil surface by priming agents such as TNFα and LPS.^64^, ^65^, ^66^ Considering the high levels of TNFα in a number of inflammatory diseases such as rheumatoid arthritis suggests that the mechanism underlying the neutrophil priming process and its consequences both in vitro and in vivo may offer new opportunities for therapeutic intervention in pathologic settings.^67^ The concentration of an FPR1 agonist needed to induce chemotaxis is commonly substantially lower than that needed to activate the superoxide NADPH‐oxidase; whereas low concentrations of RE‐04‐001, at the nM level, induced a robust activation of the NADPH‐oxidase, comparably higher concentrations were needed to induce neutrophil chemotaxis. This suggests that signaling downstream RE‐04‐001 activated FPR1 is functional selective, that is, in favor of oxidase activation over chemotaxis. To directly compare the functional selective profile of RE‐04‐001 we calculated biased signaling ratios for the agonists with the values 0.1 and 5 for RE‐04‐001 and fMLF, respectively. These values were obtained from a direct comparison of the respective EC_50_ value for activation of the NADPH‐oxidase with that to recruit neutrophils chemotactically. Thus, RE‐04‐001 clearly reveals a functional selective neutrophil response, and this was linked to a low level of β‐arrestin recruitment.

Our finding that the ability of FPR1 agonists to trigger neutrophil migration is linked to the ability to recruit β‐arrestin is in agreement with the emerging concept of biased FPR agonism and functional selectivity shown to be valid also for FPR2.^29^, ^30^, ^31^, ^45^, ^68^, ^69^ Our data are also in line with the documented role of β‐arrestin in regulating cell migration in studies performed with other cell types, including neutrophil‐like HL60 cells.^70^, ^71^, ^72^, ^73^ At the structural level, these data suggest that both FPR1 and FPR2 can be stabilized in conformations that open for one signaling pathway but not for another, a signaling bias that gives rise to a functional selective response with a downstream signaling outcome determined by the binding mode of the activating agonist. This suggestion is also supported by data obtained with variants of the prototype peptide agonist fMLF that have been shown to trigger chemotaxis but are unable to activate the ROS generating neutrophil NADPH‐oxidase.^32^ Future structural studies of FPR1 in association with different agonists should provide molecular insights into the ligand‐directed FPR1 activation mechanism. The FPR1 signaling scheme for RE‐04‐001 includes the signals that induce a transient rise in [Ca^2+^]_i_, one of the very early events in GPCR signaling, and based on the activity induced by RE‐04‐001, it is clear that this agonist is more potent than the prototype FPR1 agonist fMLF. The increase in [Ca^2+^]_i_ is not reduced by a Gαq inhibitor, and this is in line with earlier studies that have identified the βγ part of a Gαi containing G protein downstream of FPR1, to be the link between the receptor an activation of the PLC‐PIP_2_‐IP_3_‐Ca^2+^ pathway.^4^, ^43^ Similar signaling profiles of fMLF and RE‐04‐001 are obtained also for the receptor downstream signal leading to an activation of ERK1/2 phosphorylation; that is, it is clear that RE‐04‐001 is a more potent agonist than the prototype peptide agonist fMLF. Despite this, we noticed an obvious difference between the two agonists with respect to their ability to activate FPR1 for β‐arrestin recruitment, demonstrating a biased signaling profile downstream of FPR1 when activated with RE‐04‐001. Clearly, β‐arrestin does not play an essential role for RE‐04‐001‐induced FPR1 desensitization and subsequent FPR1 reactivation by latrunculin A. This drug is a well‐known actin cytoskeleton disrupting agent that by a sequestering of the free actin monomer pool in living cells, inhibits the dynamic polymerization of G‐actin,^74^ and the data obtained using latrunculin A further support the notion that FPR desensitization relies primarily on the actin cytoskeleton.^45^ We have previously demonstrated that the activities of several other neutrophil GPCRs also are regulated by the actin cytoskeleton.^31^, ^75^, ^76^

The biased signaling concept is now firmly established in GPCR biology.^27^, ^28^ Clearly, this concept is valid also for FPR1; in contrast to the prototype FPR1 agonist, RE‐04‐001 has a biased signaling profile. Similar to RE‐04‐001, several FPR2 agonists have earlier been shown to transduce a biased signaling feature in neutrophils.^29^, ^30^, ^31^ It is interesting to note that similar to RE‐04‐001, the biased signaling FPR2 agonists that lack ability to recruit β‐arrestin and are also poor neutrophil chemoattractants,^29^, ^30^, ^31^ suggesting a role for β‐arrestin in regulating both FPR1‐ and FPR2‐mediated directional cell migration. The nonpeptide compound termed Quin‐C1^77^ has also been shown to be a biased signaling FPR2 agonist, but with the reversed functional selectivity; it lacks the ability to trigger superoxide release, while being able to induce neutrophil chemotaxis.^77^ The precise mechanism that determines this type of biased signaling downstream of a receptor occupied by different ligands is not clear at present, but the molecular basis for this phenomenon has been suggested to be due to the formation of different receptor conformations induced by agonists that have different but overlapping receptor binding sites. This notion is supported by the data showing that RE‐04‐001 is unable to compete with fMLF and by that block fMLF‐induced β‐arrestin recruitment. As suggested, the background to this could be that FPR1 has two distinct binding sites that recognize fMLF and RE‐04‐001, respectively, and that the activated receptor adopts different conformations when these sites are occupied.

Regarding signaling, it should also be noticed that β‐arrestin has been suggested to regulate receptor desensitization and internalization as well as to initiate noncanonical signaling including ERK1/2 phosphorylation.^46^, ^78^ Our data showing that RE‐04‐001 at concentrations that are unable to recruit β‐arrestin potently activates the ERK1/2 phosphorylation pathway, suggesting that FPR1‐mediated ERK1/2 phosphorylation is not regulated by β‐arrestin. In addition, β‐arrestin has for many GPCRs, a key role in the process of receptor internalization, but as shown in several studies using cells deficient in β‐arrestin or FPR agonists unable to recruit β‐arrestin, the endocytic uptake of agonist occupied FPRs can occur without any involvement of β‐arrestin.^30^, ^31^, ^79^ FPR1 internalization has, however, also been suggested to be a Gαi‐independent process.^68^ It is not feasible at present to perform experiments with primary neutrophils that directly answer questions about signaling and receptor internalization, meaning that basically all results on this subject have been obtained using overexpressed receptors in nonleukocyte cell lines combined with genetic modifications and/or pharmacologic inhibitors. The results obtained in such systems do not necessarily reflect signaling/function in primary cells, as illustrated by the fact that pertussis toxin commonly used to selectively inhibit Gαi signaling is not specific for this G protein subunit when used in primary neutrophils.^43^, ^45^ Thus, results obtained from such experiments should be interpreted with caution.

Although there are differences in the activation/signaling profiles between RE‐04‐001 and the prototype peptide FPR1 agonist fMLF, our data also demonstrate that there are similarities; RE‐04‐001 similar to fMLF, interplay with other neutrophil GPCRs; this is achieved through different GPCR crosstalk mechanisms, complex phenomena with mechanisms not yet understood (see a recent review, Dahlgren et al.^45^). Nevertheless, the biologic relevance of receptor crosstalk is obvious when neutrophils facing multiple ligands that have affinity for different receptors during migration and activation process. The outcome of neutrophil activation is thus dependent on the cooperation of multiple ligands at the receptor signaling level. This cooperation is evident from our data demonstrating that RE‐04‐001 can inhibit IL‐8 but prime the PAF response. When it comes to the crosstalk between FPR1 and FFAR2, low concentrations of RE‐04‐001 could be primed by allosterically modulated FFAR2. The fact that the primed PAF response is sensitive to an FPR1 antagonist further supporting the crosstalk mechanism relies on a reactivation of desensitized FPR1.^53^, ^80^ Inflammatory mediators such as IL‐8 and PAF are potent neutrophil chemoattractants that are produced in many inflammatory settings including cystic fibrosis.^81^ Studies aiming to understand the interplay between different inflammatory mediators that regulate neutrophil functions through for example receptor crosstalk and heterologous desensitization is of importance not only to increase our knowledge about inflammation in a more complex in vivo context, but also to facilitate the design of better receptor‐based therapeutics for treatment of inflammatory diseases. With respect to the in vivo effects of FPR agonists such as RE‐04‐001, it is important to note that the receptors in man and mouse, commonly used in animal model studies, differ substantially both with respect to the ligand‐recognition profile and the number of family members across species (see a recent review on the differences in FPRs across species in Winther et al.^82^). In addition, downstream molecular mechanisms may differ between human, and mouse FPRs. A prominent example is that the potent human FPR1 agonist fMLF is a very poor agonist for its mouse orthologue, ^82^ and this indicates the possibility that even if RE‐01‐004 is a specific and biased agonist for FPR1, it is not necessarily acting the same way on mouse Fpr1. Thus, future basic characterization studies as well as studies of bioavailability and toxicity are required for an evaluation of the in vivo effects of this compound in inflammatory disease models.

In summary, we have identified and characterized RE‐01‐004, a small compound, as a potent FPR1 selective agonist, which triggers a biased FPR1 signaling leading to neutrophil functional selective response. The activation characteristics differ from the most commonly used FPR1 peptide agonist fMLF. The information provided about the basic characteristics of RE‐04‐001 is of value for further optimization processes and mechanistic studies both in vivo and in vitro and the knowledge obtained would shed light on the complex biology of FPR1 in health and in different disease conditions.

## AUTHORSHIP

H.F. and S.L. performed the experiments and analyzed the data with the input from the coauthors. H.F., C.D., R.H., P.O. designed the study and analyzed the data. H.F. and C.D. wrote the manuscript and all authors contributed to the manuscript before approving the final version.

## DISCLOSURES

P.O. and R.H. declare conflicts of interest: they are cofounders of Pronoxis AB, which has a commercial interest in the development of the FPR1 agonists such as the class of compounds represented by RE‐04‐001. The other authors declare no conflicts of interest.

## Supporting information

TableS1.docx
