# Supplementary material for: Functional selective FPR1 signaling in favor of an activation of the neutrophil superoxide generating NOX2 complex
Source: J Leukoc Biol. 2020 Oct 11;109(6):1105–20. doi: 10.1002/JLB.2HI0520-317R (PMC8246850; doi:10.1002/JLB.2HI0520-317R)
Supplement: Supplementary file 1 — TableS1.docx [file JLB-109-1105-s001.docx]

**Supplementary Table 1**

**RE-04-001 profiling using the the [HitProfilingScreen](https://www.eurofinsdiscoveryservices.com/catalogmanagement/viewitem/HitProfilingScreen-Panel/PP70) (Eurofins) panel**

| Target | Species | Family |
| --- | --- | --- |
| Adenosine A1 | Human | **GPCR** |
| Adenosine A2A | Human | **GPCR** |
| Adrenergic α2A | Human | **GPCR** |
| Adrenergic β1 | Human | **GPCR** |
| Adrenergic β2 | Human | **GPCR** |
| Cannabinoid CB1 | Human | **GPCR** |
| Dopamine D1 | Human | **GPCR** |
| Dopamine D2S | Human | **GPCR** |
| Histamine H1 | Human | **GPCR** |
| Muscarinic M2 | Human | **GPCR** |
| Muscarinic M3 | Human | **GPCR** |
| Nicotinic Acetylcholine | Human | **GPCR** |
| Nicotinic Acetylcholine α1, Bungarotoxin | Human | **GPCR** |
| Opiate μ(OP3, MOP) | Human | **GPCR** |
| Potassium Channel hERG | Human | **Ion Channel** |
| Prostanoid EP4 | Human | **GPCR** |
| Serotonin (5-Hydroxytryptamine) 5-HT2B | Human | **GPCR** |
| Sigma σ1 | Human | **Other** |
| Transporter, Norepinephrine (NET) | Human | **Transporter** |
|  |  |  |
| Adenosine A2A | Rat | **GPCR** |
| Adrenergic α1B | Rat | **GPCR** |
| Calcium Channel L-Type, Dihydropyridine | Rat | **Ion Channel** |
| GABAA, Flunitrazepam, Central | Rat | **Ion Channel** |
| GABAA, Muscimol, Central | Rat | **Ion Channel** |
| Glutamate, NMDA, Phencyclidine | Rat | **Ion Channel** |
| Imidazoline I2, Central | Rat | **Other** |
| Rolipram | Rat | **PDE** |
| Sodium Channel, Site 2 | Rat | **Ion Channel** |
|  |  |  |
| Phorbol Ester | Mouse | **PKC activator** |
|  |  |  |
| Potassium Channel [KATP] | Hamster | **Ion Channel** |

A screen panel of 30 common drug targets from different species (listed above) were used for RE-04-001 profiling.

The data show that RE-04-001 (10 µM) had neither inhibitory nor stimulatory effect (≥ 50%) on any of the targets examined.

More information about the assays for each individual target protein can be found at Eurofins homepage:

[*https://www.eurofinsdiscoveryservices.com/catalogmanagement/viewitem/HitProfilingScreen-Panel/PP70*](https://www.eurofinsdiscoveryservices.com/catalogmanagement/viewitem/HitProfilingScreen-Panel/PP70).
